# Comment on Patel, B.; Makaryus, A.N. Artificial Intelligence Advances in the World of Cardiovascular Imaging. *Healthcare* 2022, *10*, 154

**DOI:** 10.3390/healthcare10040727

**Published:** 2022-04-14

**Authors:** Daniele Giansanti

**Affiliations:** Centro Nazionale Tecnologie Innovative in Sanità Pubblica, Istituto Superiore di Sanità, 00161 Rome, Italy; daniele.giansanti@iss.it; Tel.: +39-06-49902701

Regarding Dr. Makaryus’s interesting review study [1], I would like to express my opinion on it.

I found that this work is particularly stimulating and that it gives a great deal of added value to the Special Issue “*The Artificial Intelligence in Digital Pathology and Digital Radiology: Where Are We?*” [2,3].

Specifically, I believe that this review has the great merit of focusing on the developments of Artificial Intelligence (AI) in the field of *Digital Cardiology* (DC), in a medical sector as broad and strategic as that of cardiology. Many of the considerations that emerge from the review in this specific sector of *Digital Radiology* (DR), such as those relating to the imaging, are exportable to the entire sector. From the review [1], it emerges clearly that the introduction of artificial intelligence (AI) into the world of digital cardiovascular imaging is greatly broadening the capabilities of the field, both with respect to advancements as well as with respect to the complete and accurate diagnosis of cardiovascular conditions. Among the application sectors in which the review [2] has shown the greatest potential we find recognition, diagnostics, protocol automation, and quality control for the analysis of cardiovascular imaging modalities such as, cardiovascular computed tomography, cardiovascular magnetic resonance imaging, nuclear cardiac imaging, echocardiography, and other sectors of imaging. All this is in line with what emerges in the field of DR [4] in general. All this will lead to important changes in the organization of work and to continuous challenges that will involve all actors, such as the radiologist, the hemodynamist, the cardiologist, the general practitioner, the medical radiology technician, and other professionals and patients. In DR [4], AI will be useful for: simplifying all the management activities, from the scheduling of the patients up to the reports and the bill; medical decision support in a specific imaging application; suggesting the most appropriate exam after a scrutiny of the patient’s virtual directory; both cleaning/de-noising the signal and minimizing the artifact; facilitating the automated image interpretation; and dimensional and volumetric measurements.

To support the insiders in medical activity, data science specialists work on the development of increasingly better performing and targeted algorithms that must be calibrated considering the specificity of the application, the decision-making protocols and the physical process which is different in the formation of images. For this reason, it is important that the insiders talk with these scientific scholars who are also involved in basic research both to give new stimuli and to give feedback on use. 

Precisely because of the challenges and changes taking place, in [5] it was highlighted that some studies are addressing in a targeted manner aspects relating to the acceptance and consent of the introduction of AI in DR. These studies, also reported in [5], are mainly based on questionnaires carried out in an original way, and only in rare cases are these questionnaires of a standardized type [6,7,8,9,10,11,12,13,14,15,16,17,18,19,20]. 

Among the various potentials that this type of investigation has, in addition to providing important outputs on the integration agreement of AI in DR, we find those of raising awareness among stakeholders and putting data science specialists in communication with insiders. 

These studies reported in [5] concerned all the professionals involved. The results highlighted, among other things: the importance of both looking at these professionals in a comparative and single way; to deal in a broad and detailed way with the applications of DR impacted by AI; and the need to be supported by scientific societies and by federations of scientific societies. 

I tried to see if such activities have started in the DC sector, by means of a preliminary and rapid search.

I made the following two queries on the Pubmed database:

- *Search: ((Artificial Intelligence[Title/Abstract]) AND (Cardiology[Title/Abstract])) AND (consensus [Title/Abstract])* [21].
- *Search: ((Artificial Intelligence[Title/Abstract]) AND (Cardiology[Title/Abstract])) AND (acceptance[Title/Abstract])* [22].

I found four studies; however, they did not address the issue of *acceptance* and *consensus* specifically.

I would like to ask you if you believe that, among the future work in the integration activities of AI in cardiology in the applications and sectors that you highlighted very clearly in the review, there will be a need for desirable and/or possible acceptance and consensus initiatives based on targeted investigations on insiders and, if so, if you believe that also in this case, by analogy to the DR in general, they will be based on survey tools, such as the questionnaires used in DR and with a similar approach [5].

## Data Availability

Not applicable.
